# Longitudinal associations of dynamic transitions of multidimensional health vulnerability with cardiovascular disease risk: evidence from UK Biobank

**DOI:** 10.3389/fpubh.2026.1902925

**Published:** 2026-09-22

**Authors:** Jie Wu, Linlin Chen, Siyuan Sun, Yanru Jia, Tingting Hou, Yaogeng Chen, Chenglei Zhang, Jiangping Li, Yu Zhao

**Affiliations:** 1 School of Public Health, NHC Key Laboratory of Metabolic Cardiovascular Diseases Research, Ningxia Key Laboratory of Environmental Factors and Chronic Disease Control, Ningxia Medical University, Yinchuan, Ningxia, China; 2 Department of Medical Laboratory, Cardiovascular and Cerebrovascular Disease Hospital, General Hospital of Ningxia Medical University, Yinchuan, Ningxia, China; 3 School of Medical Information and Engineering, Ningxia Medical University, Yinchuan, Ningxia, China

**Keywords:** cardiovascular disease, dynamic transitions, healthy aging, longitudinal cohort, multidimensional health vulnerability

## Abstract

**Background:**

Health vulnerability (HV), an intrinsic multidimensional construct integrating physiological, psychological, and social domains, may be associated with cardiovascular disease (CVD) risk. However, existing HV assessments lack integration and temporal dynamics change. This study identifies the dynamic association between the HV and risk of CVD.

**Methods:**

This longitudinal cohort study included 16,072 participants from the UK Biobank with HV assessments at baseline (2006–2010) and repeat assessment (2014). Latent class analysis and latent transition analysis were used to identify HV transition patterns. Cox proportional hazards models were applied to evaluate associations between HV transitions and incident CVD, with follow-up initiated from baseline assessment. Sensitivity analyses included alternative HV transitions classification, exclusion of hypertensive diseases from the composite outcome, and competing risk analyses considering non-CVD death.

**Results:**

During a median follow-up of 8.9 years, 3,033 incident CVD events were identified. Compared with persistently low vulnerability, increasing vulnerability and persistently elevated vulnerability were associated with higher CVD risk after multivariable adjustment (HR = 2.14, 95% CI: 1.74–2.63 and HR = 1.18, 95% CI: 1.08–1.29, respectively). Subgroup analyses identified significant effect modification by age and smoking status, indicating that the associations between HV transitions and CVD risk differed across these populations. The main findings remained consistent in sensitivity analyses.

**Conclusion:**

Dynamic changes in HV were associated with subsequent CVD risk, with increasing and persistently elevated vulnerability showing higher risks. These findings highlight the potential importance of monitoring multidimensional vulnerability changes for cardiovascular risk assessment.

## 1 Introduction

Cardiovascular diseases (CVD) constitute the foremost global cause of mortality and disease burden. Between 1990 and 2019, CVD prevalence surged from 271 million to 523 million cases, with annual fatalities exceeding 17 million-representing one-third of global mortality (1–3). Confronting this critical public health challenge necessitates early identification of risk factors and implementation of effective interventions to alleviate societal burdens. CVD is caused by multiple factors (4). Comprehensive assessment integrating physiological, psychological, and social dimension is essential for elucidating pathological mechanisms, thereby establishing a scientific foundation for early-phase interventions and personalized therapeutic strategies.

The conceptual framework of vulnerability originated in natural disaster research, characterizing systems' susceptibility to external perturbations and capacity for recovery (5). This construct expanded into socioeconomic domains, establishing a vulnerability systems framework for analyzing poverty, emergency responses, and analogous phenomena (6). Subsequent investigations revealed that population health systems manifest pronounced vulnerability due to synergistic interactions among natural, economic, and sociopolitical determinants, which may result in dual crises of stability and sustainability (7). Health vulnerability (HV) is defined as an intrinsic, latent biological syndrome characterized by multidimensional attributes arising from dynamic interactions among individual behavioral traits, sociocultural contexts, economic-political conditions, and lifelong biological processes (8, 9). Substantial evidence confirms significant associations between vulnerability and chronic diseases, particularly CVD, spanning disease onset, progression, and prognosis (10, 11). Pathophysiologically, hypertension and CVD share fundamental pathological bases, including chronic inflammation and metabolic dysregulation, which establishes a bidirectional vicious cycle (12). Socially, adverse relationships increase coronary heart disease incidence by 29% (13), while socioeconomic status (SES) exerts quantifiable impacts on cardiovascular health (14). Psychologically, chronic stress and anxiety disorders may increase the risk of CVD mortality (15, 16), and depression confers a 1.5-fold higher risk of coronary artery disease (17). Accordingly, these physiological-psychological-social interactions establish HV as a pivotal determinant of CVD risk.

Consequently, addressing HV has become imperative for improving population health outcomes, and rigorous assessment is essential to quantify individual health status, risks, and needs. However, current methodologies exhibit dual limitations. Firstly, the fragmentation of indicators: researchers have selected different indicators based on their own research needs, while integrated physical-mental-social assessment based on the World Health Organization (WHO) definition of health is less common (18–20). Secondly, the static perspective is insufficient, as cross-sectional designs fail to elucidate the dynamic evolution of HV and its causal relationship with CVD. To transcend these methodological constraints, our study develops a multidimensional HV index integrating physiological, psychological, and social domains and leverages longitudinal cohort data to examine the association between HV and CVD. This study provides insights into how changes in HV over time are associated with CVD risk, highlighting the potential value of monitoring vulnerability transitions for cardiovascular risk assessment and prevention.

## 2 Methods

### 2.1 Study design and population

The study used two assessment waves from the UK Biobank (UKB), a comprehensive biomedical database for population health and genetic research. Between 2006 and 2010 (baseline), more than 500,000 participants aged 40–69 years were recruited from 22 assessment centers across the United Kingdom, and repeated follow-up assessments were carried out in 2014 (follow-up assessment) (21). The study was approved by the North West Multicenter Research Ethics Committee (REC reference for UK Biobank 11/NW/0382), and all participants provided written informed consent (22). In this study, 502,369 UK adults were initially screened. Among these, we included individuals who had completed two assessments of health vulnerability (baseline and follow-up) and were free of cardiovascular disease at baseline. After exclusions (see Figure 1 for the detailed flowchart), 16,072 participants were included in the study. Detailed information regarding UK Biobank field IDs is provided in Supplementary Methods 2. This study was reported according to the Strengthening the Reporting of Observational Studies in Epidemiology (STROBE) guidelines (Supplementary Table S1).

**Figure 1:**
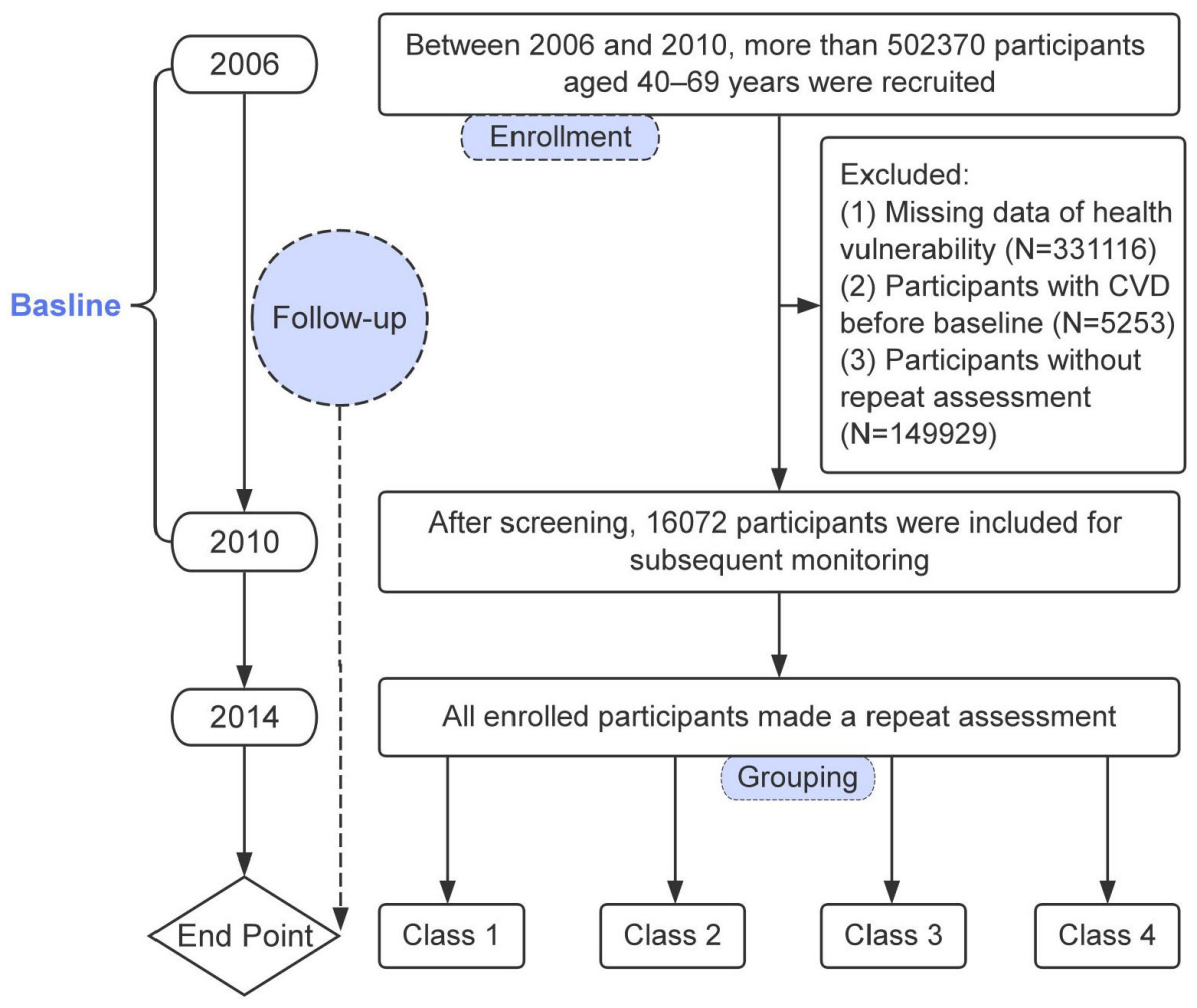
Flow chart of the study participants.

### 2.2 Assessment of health vulnerability

Researchers have adopted various methods to measure health vulnerability indicators (23), ranging from simple physiological dimensions to multidimensional concepts that incorporate both objective and subjective evaluations. For example, Wang Xuehui (24) constructed multidimensional vulnerability indicators from three dimensions: risk factors, risk resilience, and the social service system. Deng Dasong et al. (25) measured health vulnerability from two dimensions (physical and mental). However, according to WHO classification standards, residents' health encompasses multiple dimensions, including physical, mental, and social health (26). Therefore, this study operationalizes health vulnerability indicators across these three dimensions.

For the psychological dimension, we used two measures. The Eysenck Neuroticism (N-12) score (27) is calculated by assigning 1 point for a “yes” answer and 0 points for a “no” answer, with a maximum of 12 points. This neuroticism score is then divided into high and low groups using the mean score as the cut-off. For Recent Depressive Symptoms (RDS-4) (27), each symptom present is scored as 1 point (range 0–4). The physiological and social dimension indicators are listed in Table 1 (15 indicators in total). In this study, the health vulnerability index equals the sum of all 15 indicators, where a higher score corresponds to greater health vulnerability and thus worse health status (Table 1).

**Table 1:** Components of health vulnerability.

Dimension	Variables	Questions	Value
Physical	Overall health assessment	Feeling overall unhealthy?	0 = no,1 = yes
	Illness or disability	Long–term illness, disability or infirmity?	0 = no,1 = yes
	Hearing/eye problems	Do you have any difficulty with your hearing or eyes?	0 = no,1 = yes
	Falls or operations	Have you had any major operations or falls?	0 = no,1 = yes
	Pain	Is there any pain?	0 = no,1 = yes
	physical activity	Are you physically inactive?	0 = no,1 = yes
Mental	N−12	Mood swings	0 = low,1 = high
		Miserableness	
		Irritability	
		Sensitivity/hurt feelings	
		Fed–up feelings	
		Nervous feelings	
		Worrier/anxious feelings	
		Tense	
		Worry too long after embarrassment	
		Suffer from “nerves”	
		Loneliness, isolation	
		Guilty feelings	
	RDS-4	Frequency of depressed mood in last 2 weeks	0 = no,1 = yes
		Frequency of unenthusiasm/disinterest in last 2 weeks	
		Frequency of tenseness/restlessness in last 2 weeks	
		Frequency of tiredness/lethargy in last 2 weeks	
	Seen doctor	Seen doctor (GP) for nerves, anxiety, tension and depression?	0 = no,1 = yes
	Health satisfaction	You are dissatisfied with your health?	0 = no,1 = yes
	Happiness	You feel unhappy?	0 = no,1 = yes
Social	Number in household	Do you live alone?	0 = no,1 = yes
	Friend/family visits	Family and friends rarely visit?	0 = no,1 = yes
	Able to confide	No one close to me to confide?	0 = no,1 = yes
	Leisure/social activities	Not engage in social leisure activities?	0 = no,1 = yes

In the UK Biobank, the baseline assessment of health vulnerability was conducted during recruitment (2006–2010), and the follow-up assessment was performed at a repeat visit in 2014. Cumulative health vulnerability (CHV) was calculated by the average of the two assessment scores multiplied by the time interval (in years) between the two follow-up:


$$\begin{array}{l} \text{CHV}=\left[\left({\text{HV}}_{\text{baseline}}+{\text{HV}}_{\text{follow}-\text{up}}\right)/2\right]\times \text{time interval} \end{array}$$ (1)

here *HV*_baseline_ and HV_follow − up_ are the HV scores in baseline and follow-up respectively.

### 2.3 Definitions of outcomes

The primary outcome was incident CVD, defined as the first occurrence of ischemic heart disease (including angina pectoris, myocardial infarction, chronic ischemic heart disease, and heart failure; ICD-10 codes: I20–I25 and I50), atrial fibrillation (ICD-10: I48), hypertensive (ICD-10: I10), and other cardiovascular diseases, including peripheral artery disease, stroke, hypertensive heart disease, pulmonary heart disease, cardiomyopathy, myocarditis, and rheumatic heart disease (ICD-10 codes within I00–I99 according to specific conditions).

Participants with pre-existing CVD or hypertension at baseline were excluded, whereas incident CVD events occurring between baseline and the repeat HV assessment were retained in the follow-up analyses, as survival time was calculated from the baseline assessment date. Person-years of follow-up were calculated from the date of the baseline assessment (2006–2010) until the date of first CVD diagnosis, death, loss to follow-up, or the end of follow-up (2 November 2022), whichever occurred first.

### 2.4 Covariates

The following baseline potential confounders were included in the analysis: age, sex (male or female), education level (less than college or college or above), smoking status (never, former, or current), drinking status (never, former, or current), body mass index (BMI), diabetes, race (white, or non-white), CVD family history, and lipid profile parameters, including total cholesterol (TC), triglycerides (TG), high-density lipoprotein cholesterol (HDL-C), and low-density lipoprotein cholesterol (LDL-C).

### 2.5 Statistical analysis

Latent class analysis (LCA) was conducted separately at baseline (2006–2010) and follow-up (2014) using Mplus version 8.3 to identify unobserved subgroups with similar patterns of health vulnerability indicators. Models with one to five latent classes were fitted, and the optimal solution was determined based on log-likelihood, AIC, BIC, aBIC, entropy, LMR-LRT, class size, and clinical interpretability (28). Significant LMR-LRT results indicated that models with additional classes generally provided improved statistical fit compared with models with one fewer class. Entropy values around 0.60–0.80 were considered indicative of acceptable classification quality (29). Although models with four and five classes showed lower AIC, BIC, and aBIC values, these solutions showed reduced entropy and limited improvement in clinical interpretability compared with the three-class solution. Considering model fit, classification quality, class size, and clinical interpretability, the three-class solution was selected as the optimal model at both baseline and follow-up (Table 2). The identified classes were characterized as high, moderate, and low health vulnerability groups based on item-response probabilities of the 15 indicators (Supplementary Table S3). Posterior classification probabilities and average latent class assignment probabilities indicated acceptable class separation at both baseline and follow-up (Supplementary Table S4). These latent classes were subsequently used in latent transition analysis (LTA) to estimate changes in vulnerability status between baseline and follow-up. Detailed item-response probabilities, classification probabilities, and frequencies of the original latent transition patterns are presented in Supplementary Tables S3-S4, Table 3.

**Table 2:** Model fit indices and classification quality of latent class models at baseline and follow–up.

Time	Classes	df	Loglikelihood	AIC	BIC	aBIC	Entropy	LMR(P)
Baseline	1	15	−104,017.4	208,064.8	208,180.1	208,132.4		
	2	31	−100,267.1	200,596.2	200,834.5	200,735.9	0.645	< 0.001
	3	47	−99,581.5	199,257.0	199,618.2	199,468.9	0.732	< 0.001
	4	63	−99,284.0	198,694.0	199,178.2	198,977.9	0.630	< 0.001
	5	79	−99,153.2	198,464.5	199,071.6	198,820.5	0.584	< 0.001
Follow–up	1	15	−107,314.8	214,659.5	214,774.8	214,727.1		
	2	31	−103,277.9	206,617.7	206,855.9	206,757.4	0.648	< 0.001
	3	47	−102,518.8	205,131.7	205,492.8	205,343.5	0.712	< 0.001
	4	63	−102,150.9	204,427.8	204,911.9	204,711.8	0.667	< 0.001
	5	79	−101,988.7	204,135.3	204,742.4	204,491.4	0.634	< 0.001

**Table 3:** Distribution of the nine original latent transition patterns and their corresponding longitudinal vulnerability categories.

Baseline state	Trends	Follow–up State	Longitudinal vulnerability pattern	*n*	Overall transition (%)	Transition within baseline type (%)
High (*n* = 2,226)	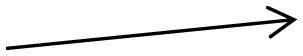	High	Class 4	2,185	13.60	98.16
	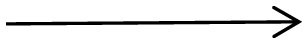	Moderate	Class 2	2	0.01	0.09
	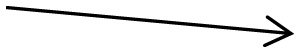	Low	Class 2	39	2.43	1.75
Moderate (*n* = 4,885)	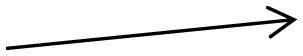	High	Class 3	43	2.68	0.88
	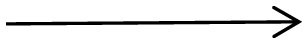	Moderate	Class 4	4,745	29.52	97.13
	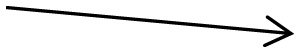	Low	Class 2	97	0.60	1.99
Low (*n* = 8,961)	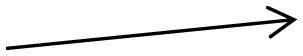	High	Class 3	138	0.86	1.54
	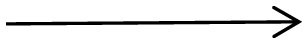	Moderate	Class 3	133	0.83	1.48
	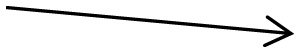	Low	Class 1	8,690	54.07	96.98

Overall transition (%) = *n*/12,882 × 100%.

Transition within baseline type (%) = *n*/baseline class size × 100%.

Latent transition analysis (LTA) was subsequently performed to examine changes in health vulnerability status between baseline and follow-up. The LTA model estimated transition probabilities between the three latent classes identified at each assessment, resulting in nine possible transition patterns between baseline and follow-up vulnerability states. The aggregation of these transition patterns was not based solely on statistical considerations but also on clinical interpretability, with the aim of capturing meaningful patterns of vulnerability change while maintaining sufficient sample size for reliable estimation. To facilitate clinical interpretation, these nine transition patterns were grouped into four longitudinal vulnerability patterns according to the direction and persistence of vulnerability change. Class1: Persistently low vulnerability, defined as remaining in the low vulnerability class at both assessments; Class2: Decreasing vulnerability, defined as transitioning from a higher vulnerability state (moderate or high vulnerability) to a lower vulnerability state; Class3: Increasing vulnerability, defined as transitioning from a lower vulnerability state (low or moderate vulnerability) to a higher vulnerability state; Class4: Persistently elevated vulnerability, defined as remaining in moderate or high vulnerability states over time. All other analyses were completed in R version 4.4.2.

Baseline characteristics were presented as percentages for categorical variables, as means ± standard deviations for normally distributed continuous variables, and as medians with interquartile ranges for non-normally distributed variables. Kaplan-Meier cumulative incidence plots were generated to assess the association between changes in HV (or CHV) and CVD events during follow-up. Three Cox proportional hazards models were fitted to investigate the longitudinal association between health vulnerability dynamics and the risk of developing CVD. Model 1 was unadjusted. Model 2 was adjusted for age and sex. Model 3 was adjusted for the covariates in Model 2 plus education level, smoking status, drinking status, BMI, race, CVD family history, diabetes, and blood lipids (TC, TG, HDL-C, LDL-C). The proportional hazards assumption was assessed using Schoenfeld residual tests based on the cox.zph function in R. The assumption was evaluated for all Cox regression models, including unadjusted, minimally adjusted, and fully adjusted models. The corresponding results are presented in Supplementary Methods 3 and Supplementary Table S5. When evidence of proportional hazards violations was observed, variable-specific Schoenfeld residual tests were examined to identify the covariates responsible for the violations. Sensitivity analyses using stratified Cox models were then performed by stratifying on covariates that violated the proportional hazards assumption, while retaining the primary exposure variables and other covariates in the models. BMI was categorized at 24 kg/m^2^ for stratification. The proportional hazards assumption was reassessed in the stratified models.

Restricted cubic spline (RCS) was used to assess whether there was a non-linear association between cumulative health vulnerability and CVD. In subgroup analyses, the effects of sex, age, education, diabetes, smoking, drinking, BMI, race, CVD family history, and blood lipids on the relationship between changes in HV and CVD risk were further examined. Multiplicative interaction terms were fitted to examine potential effect modification by these subgroup variables. Participants with missing HV indicators were excluded because complete information was required to calculate the cumulative health vulnerability index and define HV transition patterns. No participants were excluded based on missing covariates, and no prespecified threshold for participant-level covariate missingness was applied. Missing covariates were handled using multiple imputation by chained equations (MICE) with predictive mean matching (PMM), generating five imputed datasets under the missing-at-random assumption. Estimates from the five imputed datasets were combined using Rubin's rules. Survival time and CVD event status were included as auxiliary variables in the imputation model, whereas the primary exposure variables were not imputed. Detailed information on the extent of missing data, imputation model, and diagnostic procedures is provided in Supplementary Methods 1.

### 2.6 Sensitivity analyses

Sensitivity analyses were performed to assess the robustness of the main findings. First, to evaluate whether the inclusion of hypertensive diseases influenced the observed associations, we repeated the analyses using a modified composite CVD outcome excluding hypertensive diseases. The same analytical cohort and exclusion criteria as the primary analysis were retained, with only the outcome definition modified. Second, to address potential temporal misclassification and immortal time bias arising from the ascertainment of HV transition status at the repeat assessment, we performed an additional landmark sensitivity analysis. Participants who developed CVD between the baseline and repeat HV assessments were excluded, and the date of the repeat HV assessment in 2014 was used as the time origin for subsequent CVD follow-up. Among the 16,072 participants in the analytical cohort, 3,190 (19.9%) developed CVD or hypertension between the baseline and repeat HV assessments and were excluded from this sensitivity analysis. Third, to evaluate the robustness of HV transition classification, the original four-class transitions were reclassified into three broader patterns by combining Class 2 (decreasing vulnerability) and Class 3 (increasing vulnerability) as dynamic vulnerability, yielding three groups: stable low vulnerability, dynamic vulnerability, and persistent elevated vulnerability. The associations with CVD risk were subsequently reassessed. Fourth, to address potential violations of the proportional hazards assumption, stratified Cox sensitivity analyses were performed by stratifying on sex, education, race, BMI category, and smoking status. BMI was categorized at 24 kg/m^2^ for stratification. The models were adjusted for age, drinking status, diabetes status, lipid profiles (TC, TG, HDL, and LDL), and CVD family history, and the proportional hazards assumption was reassessed using Schoenfeld residual tests. Finally, competing risk analyses were conducted using the Fine-Gray subdistribution hazard model, considering death from causes other than CVD as a competing event. All sensitivity analyses were performed using the fully adjusted Cox regression model, with the same covariates as in the primary analysis.

## 3 Results

The final analytical sample included 16,072 participants (55.1% female, mean baseline age = 54.88 ± 7.61 years). Table 4 presents the baseline characteristics of the participants across the four classes of health vulnerability. The prevalence of Class 1, Class 2, Class 3, and Class 4 was 8,690, 138, 314, and 6,930, respectively. Notably, Class 4 (persistently elevated level) had the highest proportion of women (63.8%) and higher prevalence of diabetes (1.8%) than Class 1 (1.3%, *P* = 0.003). Class 3, despite its small size, showed the highest prevalence of diabetes (3.2%) and current smoking (7.6%). In contrast, Class 1 had the lowest current smoking rate (5.4%) and optimal metabolic profiles, even though this group was older.

**Table 4:** Baseline characteristics of participants stratified by class of health vulnerability.

Characteristics	Overall	Class 1	Class 2	Class 3	Class 4	*P* value
**Age, years**	54.88 ± 7.61	55.89 ± 7.53	53.38 ± 6.85	53.68 ± 7.70	53.71 ± 7.53	< 0.001
**Sex**, ***n*** **(%)**						< 0.001
Male	7,217 (44.9)	4,489 (51.7)	69 (50.0)	151 (48.1)	2,508 (36.2)	
Female	8,855 (55.1)	4,201 (48.3)	69 (50.0)	163 (51.9)	4,422 (63.8)	
**Education**, ***n*** **(%)**						0.001
Below	3,947 (24.6)	2,027 (23.3)	34 (24.6)	78 (24.8)	1,808 (26.1)	
Higher	12,125 (75.4)	6,663 (76.7)	104 (75.4)	236 (75.2)	5,122 (73.9)	
**Smoking**, ***n*** **(%)**						< 0.001
Never	10,043 (62.5)	5,553 (63.9)	86 (62.3)	199 (63.4)	4,205 (60.7)	
Former	5,003 (31.1)	2,670 (30.7)	45 (32.6)	91 (29.0)	2,197 (31.7)	
Current	1,026 (6.4)	467 (5.4)	7 (5.1)	24 (7.6)	528 (7.6)	
**Alcohol intake**, ***n*** **(%)**						< 0.001
Never	439 (2.7)	227 (2.6)	5 (3.6)	13 (4.1)	194 (2.8)	
Former	334 (2.1)	136 (1.6)	5 (3.6)	7 (2.2)	186 (2.7)	
Current	15,299 (95.2)	8,327 (95.8)	128 (92.8)	294 (93.6)	6,550 (94.5)	
**BMI, kg/m** ^**2**^	26.12 ± 3.98	26.03 ± 3.73	25.70 ± 4.30	26.39 ± 4.15	26.23 ± 4.26	0.006
**TC, mmol/L**	5.84 ± 1.05	5.86 ± 1.05	5.69 ± 0.99	5.72 ± 1.06	5.81 ± 1.06	0.001
**TG, mmol/L**	1.61 ± 0.95	1.61 ± 0.93	1.51 ± 0.94	1.62 ± 1.10	1.62 ± 0.97	0.554
**HDL–C, mmol/L**	1.52 ± 0.38	1.51 ± 0.39	1.54 ± 0.37	1.50 ± 0.38	1.52 ± 0.38	0.612
**LDL–C, mmol/L**	3.65 ± 0.81	3.67 ± 0.81	3.50 ± 0.75	3.55 ± 0.81	3.62 ± 0.81	< 0.001
**Diabetes**, ***n*** **(%)**						0.003
Yes	247 (1.5)	110 (1.3)	1 (0.7)	10 (3.2)	126 (1.8)	
No	15,825 (98.5)	8,580 (98.7)	137 (99.3)	304 (96.8)	6,804 (98.2)	
**Race**, ***n*** **(%)**						0.502
Non–white	746 (4.6)	422 (4.9)	7 (5.1)	12 (3.8)	305 (4.4)	
White	15,326 (95.4)	8,255 (95.1)	146 (94.9)	312 (96.2)	6,613 (95.6)	
**CVD family history**, ***n*** **(%)**						0.211
Yes	8,616 (53.6)	4,611 (52.8)	95 (56.0)	182 (49.7)	3,728 (54.0)	
No	7,456 (46.4)	4,076 (47.2)	69(44.0)	164 (50.3)	3,157 (46.0)	

Values are presented as mean ± standard deviation for continuous variables and number (percentage) for categorical variables. *P* values were calculated using ANOVA or chi-square tests, as appropriate.

The Kaplan-Meier estimated cumulative incidence curves depicted in Figure 2 illustrate the relationship between HV categories (A), CHV scores (B), and the risk of incident CVD. A graded association was observed between health vulnerability categories and CVD risk. Specifically, Class 1 (persistently low vulnerability) showed the lowest cumulative incidence of CVD throughout follow-up, whereas Class 4 (persistently elevated vulnerability) demonstrated the highest cumulative incidence, with increasing separation from Class 1 after approximately 4,000 days.

**Figure 2:**
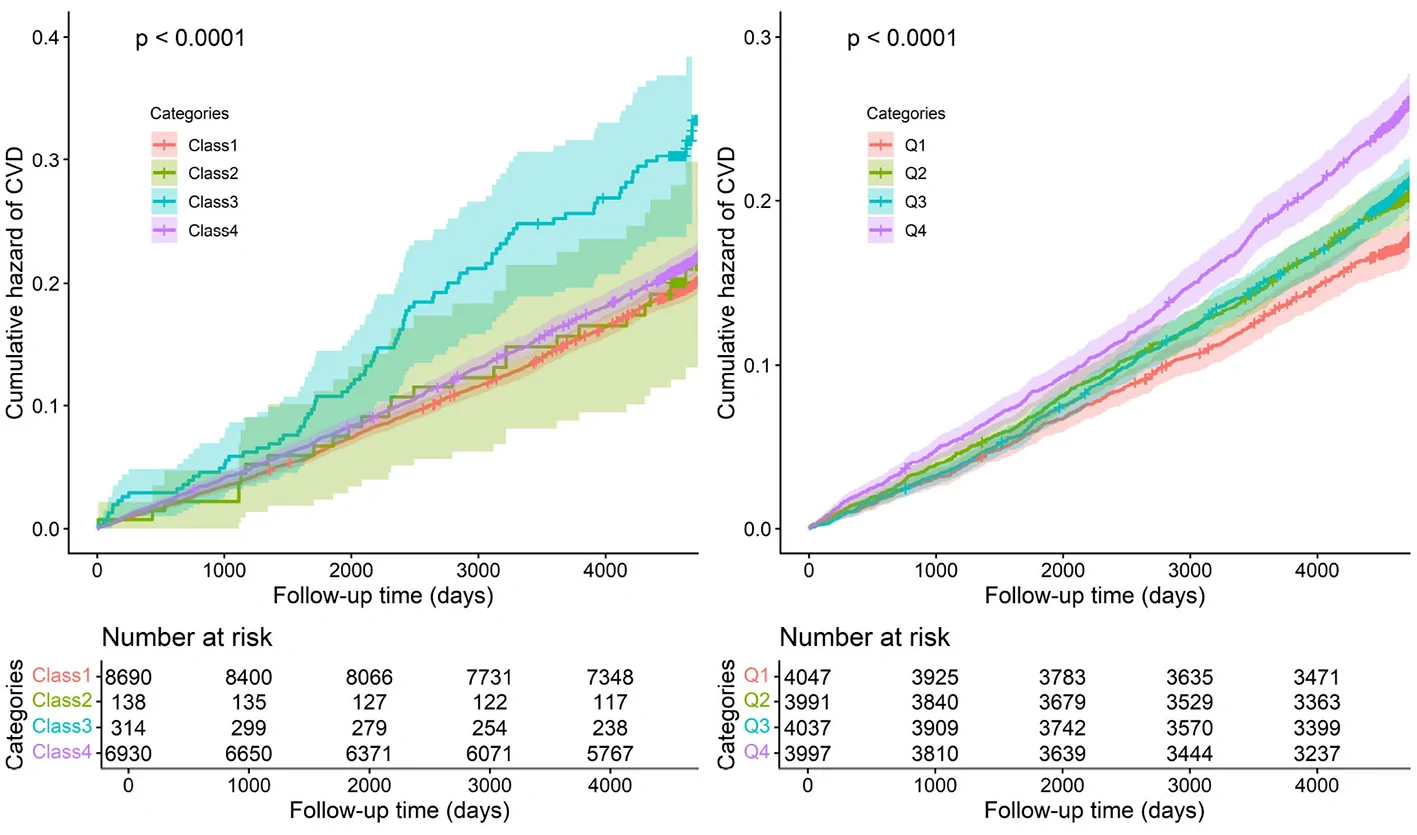
Kaplan-Meier estimated cumulative incidence curves of incident CVD according to HV categories and CHV scores, **(A)** For categories **(B)** For quartiles.

As CHV increased from Q1 to Q4, the cumulative incidence of CVD progressively increased, with the Q4 group showing a significantly higher incidence than the Q1 group (log-rank *P* < 0.001). The difference between groups became more evident during extended follow-up. Overall, higher health vulnerability was associated with a greater cumulative incidence of CVD (*P* < 0.001), suggesting an increasing accumulation of cardiovascular risk over time. Individuals with persistently elevated vulnerability (Class 4/Q4) experienced a higher burden of incident CVD during follow-up.

Over a median follow-up period of 8.9 years, 3,033 first CVD events (18.9%) were recorded. Table 5 shows the association between CHV categories and incident CVD risk, including 559 cases of ischemic heart disease, 294 cases of atrial fibrillation, 1,889 cases of hypertensive heart disease, and 298 cases of other cardiovascular diseases. In comparison with Class 1, participants in Class 3 had a significantly higher risk of incident CVD (HR = 1.79, 95% CI: 1.43–2.22, *P* < 0.001), and Class 4 was also associated with an increased risk of CVD (HR = 1.28, 95% CI: 1.19–1.38, *P* < 0.001) in Model 3. However, no significant association was observed between Class 2 and CVD risk (HR = 1.22, 95% CI: 0.82–1.82, *P* = 0.320). Trend tests indicated a significant increase in CVD risk with increasing HV categories (*P* < 0.001).

**Table 5:** Associations of different health vulnerability classes with CVD.

Variables	Case, *n* (%)	Model 1^a^		Model 2^b^		Model 3^c^	
		HR (95% CI)	*P* value	HR (95% CI)	*P* value	HR (95% CI)	*P* value
Categories							
Class 1	1,563 (51.6)	1.00 (Ref)		1.00 (Ref)		1.00 (Ref)	
Class 2	26 (0.9)	1.05 (0.71–1.55)	0.805	1.23 (0.83–1.81)	0.303	1.22 (0.82–1.82)	0.320
Class 3	86 (2.8)	1.62 (1.31–2.02)	< 0.001	1.83 (1.47–2.27)	< 0.001	1.79 (1.43–2.22)	< 0.001
Class 4	1,358 (44.7)	1.10 (1.02–1.18)	0.009	1.33 (1.23–1.43)	< 0.001	1.28 (1.19–1.38)	< 0.001
***P*** **for trend**			0.005		< 0.001		< 0.001
**Cumulative health vulnerability**		1.15 (1.11–1.19)	< 0.001	1.20 (1.16–1.24)	< 0.001	1.15 (1.12–1.20)	< 0.001
Quartiles							
Q1 (< 19.0)	645 (21.3)	1.00 (Ref)		1.00 (Ref)		1.00 (Ref)	
Q2 (19.0–30.1)	727 (23.9)	1.16 (1.04–1.29)	0.007	1.18 (1.06–1.32)	0.002	1.13 (1.01–1.26)	0.028
Q3 (30.1–44.2)	756 (24.9)	1.18 (1.07–1.32)	0.002	1.27 (1.15–1.41)	< 0.001	1.21 (1.09–1.35)	< 0.001
Q4 (≥44.2)	905 (29.9)	1.46 (1.32–1.62)	< 0.001	1.64 (1.48–1.82)	< 0.001	1.47 (1.32–1.63)	< 0.001
***P*** **for trend**			< 0.001		< 0.001		< 0.001

**a Model 1** unadjusted.

**b Model 2** adjusted for age, sex.

**c Model 3** further adjusted for education level, smoking status, drinking status, diabetes status, race, CVD family history, BMI, TG, TC, HDL, LDL on model 2.

When CHV scores were categorized into quartiles, participants in the highest quartile (Q4) had a significantly higher risk of incident CVD compared with those in the lowest quartile (Q1) in Model 3 (HR = 1.47, 95% CI: 1.32–1.63, *P* < 0.001). Trend tests also showed a significant increase in CVD risk with higher CHV (*P* < 0.001).

Table 6 presents the associations between HV categories and specific CVD subtypes. Compared with Class 1, no significant associations were observed between HV categories and the risks of atrial fibrillation, ischemic heart disease, hypertensive heart disease, or other cardiovascular diseases after adjustment. Similarly, no significant associations were observed for other CVD subtypes across different HV categories. The lack of statistically significant associations for individual CVD subtypes may partly reflect reduced statistical power due to smaller numbers of subtype-specific events.

**Table 6:** Associations between health vulnerability categories and risks of specific CVD subtypes.

CVD type	Class 1	Class 2		Class 3		Class 4	
		HR (95% CI)	*P* value	HR (95% CI)	*P* value	HR (95% CI)	*P* value
**AF**	1.00 (Ref)	0.38 (0.05–2.75)	0.340	1.16 (0.57–2.39)	0.678	0.86 (0.67–1.1)	0.234
**IHD**	1.00 (Ref)	0.6 (0.19–1.87)	0.377	1.27 (0.78–2.06)	0.332	1.01 (0.84–1.21)	0.923
**HTN**	1.00 (Ref)	1.08 (0.67–1.76)	0.744	0.99 (0.73–1.33)	0.933	1.08 (0.98–1.19)	0.131
**Others**	1.00 (Ref)	1.6 (0.59–4.34)	0.358	1.74 (0.95–3.17)	0.071	0.98 (0.77–1.26)	0.892

**AF**, atrial fibrillation; **IHD**, ischemic heart disease; **HTN**, hypertension.

Class 1 served as the reference group. Hazard ratios were estimated separately for each CVD subtype.

Furthermore, multivariate-adjusted restricted cubic spline analyses demonstrated a significant linear positive association between CHV and CVD risk, while no evidence of a significant nonlinear relationship was observed (*P* for nonlinearity = 0.363; *P* for linearity < 0.001). These findings suggest that increasing CHV is associated with a continuously elevated risk of CVD (Figure 3).

**Figure 3:**
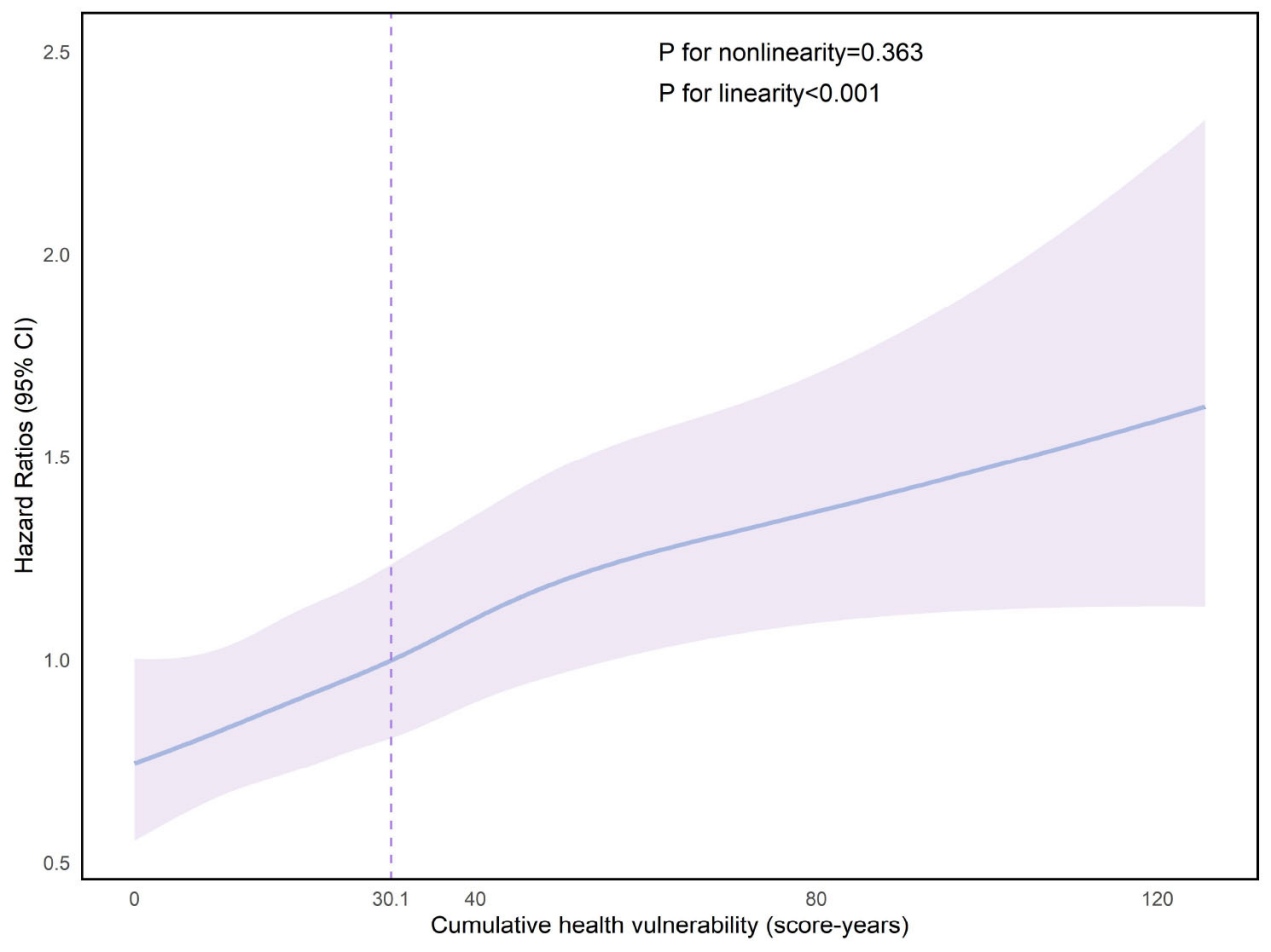
Associations of cumulative health vulnerability with risk of CVD.

Subgroup analyses were performed to assess potential effect modification of the association between HV transitions and CVD risk. Significant interactions were observed for age (*P* for interaction = 0.036) and smoking status (*P* for interaction = 0.002). Age modified the association between HV transitions and CVD risk. Among participants aged < 60 years, Class 3 showed the highest CVD risk (HR = 2.12, 95% CI: 1.61–2.78), whereas among those aged ≥60 years, Class 4 remained significantly associated with increased CVD risk (HR = 1.14, 95% CI: 1.02–1.28). Smoking status also influenced the association between HV transitions and CVD risk, with Class 3 showing the highest risk among current smokers (HR = 3.04, 95% CI: 1.58–5.87). No significant interactions were observed for other examined subgroups. Although BMI did not significantly modify the association, participants with BMI ≥24 kg/m^2^ showed increased CVD risks for Class 3 (HR = 1.83, 95% CI: 1.44–2.33) and Class 4 (HR = 1.24, 95% CI: 1.14–1.35). Overall, Class 4 remained consistently associated with elevated CVD risk across most subgroups (Figure 4).

**Figure 4:**
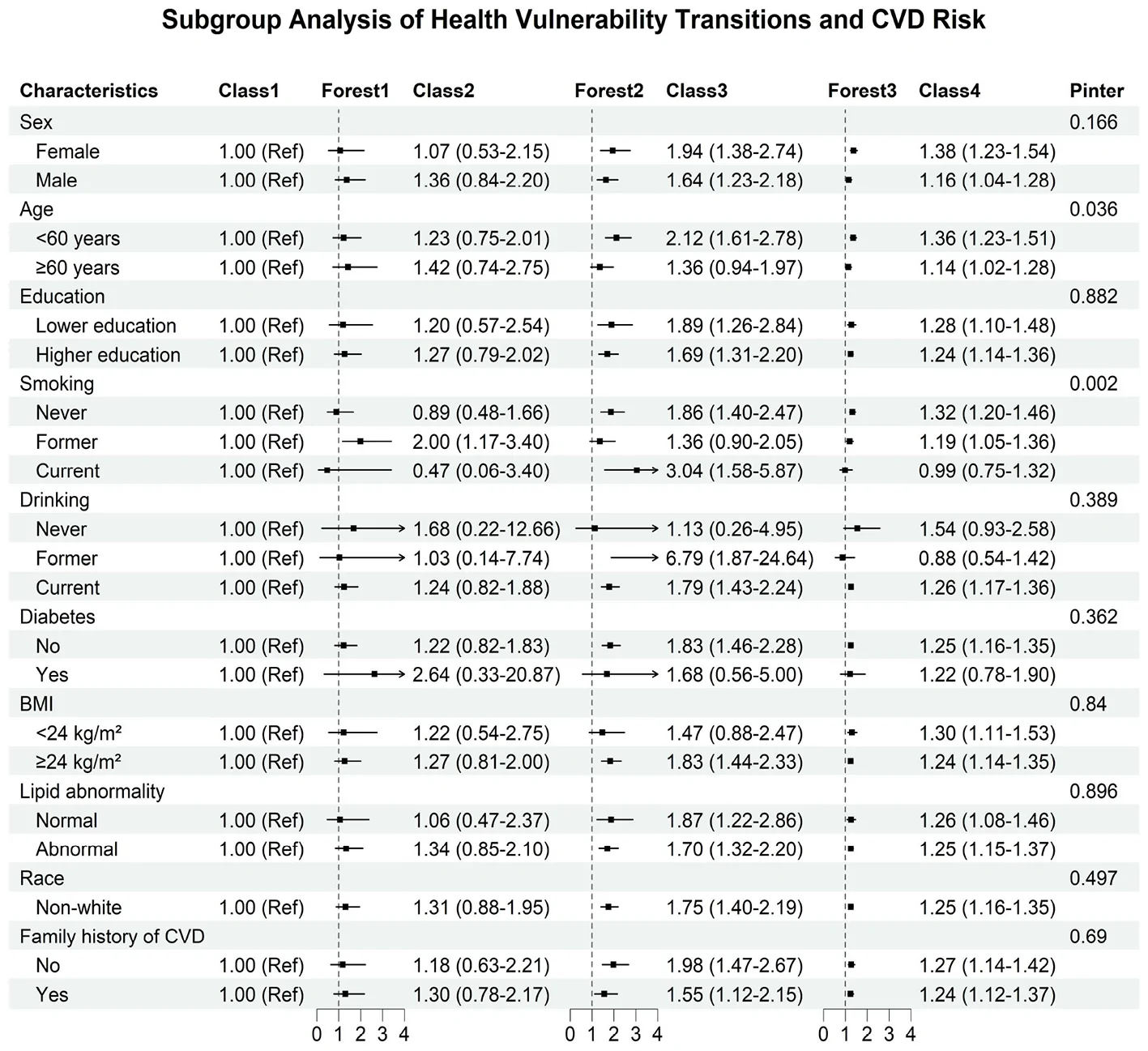
Subgroup and interaction analyses among the four classes of HV and CVD.

Sensitivity analyses yielded consistent results. First, after excluding hypertensive diseases from the composite CVD outcome, Class 3 (increasing vulnerability) and Class 4 (persistently elevated vulnerability) remained associated with higher CVD risk compared with Class 1 (HR = 2.14, 95% CI: 1.54–2.97 and HR = 1.18, 95% CI: 1.05–1.34, respectively), whereas the association for Class 2 was not statistically significant (Supplementary Table S7). Similarly, CHV remained positively associated with CVD risk (HR per 1-SD increase = 1.13, 95% CI: 1.07–1.20). Second, after excluding the 3,190 participants who developed CVD between the baseline and repeat HV assessments and initiating follow-up at the 2014 repeat assessment, the associations between HV transition categories and subsequent CVD risk remained consistent with the primary analysis (Supplementary Table S8). Compared with Class 1, persistent elevated vulnerability (Class 4) remained associated with higher CVD risk (HR = 1.18, 95% CI: 1.02–1.36), while the associations for other HV transition categories were not statistically significant. Similarly, CHV remained positively associated with CVD risk (HR per 1-SD increase = 1.23, 95% CI: 1.14–1.33). These findings suggest that the observed associations were unlikely to be driven primarily by cardiovascular events occurring before HV transition classification. Third, reclassification of HV transitions into three broader categories by combining Class 2 and Class 3 as dynamic vulnerability produced similar results (Supplementary Table S6). Compared with stable low vulnerability, both dynamic vulnerability (HR = 1.62, 95% CI: 1.33–1.96) and persistent elevated vulnerability (HR = 1.28, 95% CI: 1.19–1.38) were associated with higher CVD risk (both *P* < 0.001). The direction and magnitude of these associations were consistent with the primary four-class analysis, and the overall conclusions remained unchanged. Fourth, to address potential violations of the proportional hazards assumption, stratified Cox models were fitted by stratifying on sex, education, race, BMI category, and smoking status. After stratification, no evidence of violation of the proportional hazards assumption was observed in Model 2 (global *P* = 0.203) or in Model 3 for the HV transition, cumulative HV, or CHV quartile models (global *P* = 0.422, 0.278, and 0.397, respectively; Supplementary Table S11). The associations remained largely consistent with those from the primary analyses. Finally, Fine-Gray competing risk analyses yielded similar results, with elevated CVD risk observed for Class 3 (HR = 1.77, 95% CI: 1.42–2.22) and Class 4 (HR = 1.30, 95% CI: 1.21–1.41), supporting the robustness of the primary findings after accounting for competing mortality risk.

## 4 Discussion

This study highlights the association between HV transitions and CVD risk within a multidimensional vulnerability framework. Increasing and persistently elevated vulnerability were associated with higher CVD risk compared with persistently low vulnerability. Furthermore, the linear association between cumulative HV and CVD risk suggests that higher vulnerability burden is consistently associated with elevated cardiovascular risk. These findings indicate the potential value of HV assessment for characterizing health status and identifying individuals with higher cardiovascular risk.

Several mechanisms may explain this association. From a physiological perspective, among physically impaired populations, functional limitations and activity restrictions, combined with environmental barriers, may lead to sedentary lifestyles. This, in turn, can cause a decline in basal metabolic rate, positive energy balance, and alterations in body composition, contributing to progressive loss of muscle mass and increased adipose tissue, which heighten the risk of developing CVD (30, 31).

From psychological and social perspectives, the association of mental and social vulnerability with increased CVD risk has been demonstrated. This association may be explained by neuroendocrine imbalances, autonomic dysfunction, and elevated systolic blood pressure. These factors can trigger an inflammatory response and increase long-term inflammatory load, which adversely affects cardiovascular health. Research has shown that most forms of psychological distress (e.g., anger, anxiety, depression, and post-traumatic stress disorder), as well as social isolation and loneliness, activate the hypothalamic-pituitary-adrenal axis and the sympathetic nervous system. This leads to enhanced inflammation, oxidative stress, and autonomic dysregulation. These changes can trigger atherosclerosis and elevated systolic blood pressure, which have been associated with an increased risk of hypertension, atrial fibrillation, atherosclerosis, and other CVDs (32, 33).

We found that increasing vulnerability was associated with an even higher risk than persistently elevated vulnerability, although both groups showed elevated risk relative to persistently low vulnerability. However, the association with CVD was stronger in those with increasing vulnerability than in those with persistently elevated vulnerability. This may be due to a dramatic deterioration in health may have more pronounced effects on the cardiovascular system, such as increased chronic inflammation, exacerbation of metabolic disorders, or sudden physiological stress due to disease progression. The association with CVD was less pronounced in those with high level decreasing, possibly because the process of improving health vulnerability is longer and does not fully offset the previously accumulated health burden within the follow-up period, or because individuals remain exposed to certain risk factors during the process of improvement.

In analyses of specific CVD subtypes, no significant associations were observed between HV categories and the risks of atrial fibrillation, ischemic heart disease, hypertensive heart disease, or other cardiovascular diseases after multivariable adjustment (Table 6). These findings may reflect limited statistical power due to the smaller number of subtype-specific events. Further studies with larger sample sizes are needed to determine whether HV differentially influences specific cardiovascular disease pathways.

Our subgroup analyses suggested that age and smoking status may modify the association between HV transitions and CVD risk. Among younger adults (< 60 years), the increasing vulnerability (Class 3) showed the highest CVD risk, suggesting that early deterioration in health vulnerability may represent an important window for preventive intervention. Targeting modifiable risk factors before substantial vulnerability accumulation may help reduce future CVD risk in younger populations (34). In contrast, among older adults (≥60 years), persistent elevated vulnerability (Class 4) was associated with increased CVD risk, consistent with previous evidence showing that frailty progression and sustained frailty are strongly linked to cardiovascular outcomes (35). Therefore, long-term management of persistent vulnerability may be particularly important in older adults, as improvement or recovery from frailty has been associated with reduced CVD risk (36).

Smoking may further amplify the adverse cardiovascular consequences associated with worsening vulnerability. Among current smokers, the increasing vulnerability (Class 3) showed the greatest excess CVD risk, highlighting the potential synergistic effects of smoking exposure and vulnerability accumulation. These findings emphasize the importance of smoking cessation combined with early vulnerability management as complementary strategies for CVD prevention.

Although BMI category did not significantly modify the association between HV transitions and CVD risk, higher BMI was associated with increased CVD risk across vulnerability transitions. This may reflect shared pathways involving metabolic burden, chronic inflammation, and metabolic dysregulation linking obesity, vulnerability, and cardiovascular disease (37)^.^ Previous studies have shown that individuals with both frailty and metabolic disorders, including central obesity, experience substantially increased CVD mortality risk (38). Therefore, multidomain lifestyle interventions targeting metabolic health and vulnerability-related deficits may provide cardiovascular benefits, particularly before extensive health deterioration occurs (39).

Previous studies have mainly focused on static health vulnerability, whereas this study employed a longitudinal LCA approach to characterize temporal changes in health vulnerability patterns, highlighting the importance of considering vulnerability changes over time (40–42). LCA is regarded as a statistically robust clustering method that enables the classification of populations into distinct subgroups based on shared characteristics of health vulnerability (43). Furthermore, LTA was applied to examine transitions between latent classes over time, providing additional insights into heterogeneity in vulnerability patterns and associated cardiovascular risks. This approach may contribute to a better understanding of population-level vulnerability patterns and inform future research on cardiovascular risk assessment and prevention.

Despite its strengths, several limitations should be acknowledged. First, HV transitions were derived from only two assessment time points, baseline and repeat assessment. This may limit our ability to capture detailed longitudinal changes and identify optimal intervention windows. Although CVD follow-up in the primary analysis started at baseline, HV transition status could only be determined after the repeat assessment. Therefore, CVD events occurring between the two assessments may have preceded transition classification, potentially introducing temporal misclassification and immortal time bias. To address this concern, we performed a landmark sensitivity analysis using the 2014 repeat assessment as the time origin and excluding participants who developed CVD or hypertension between the two assessments. The results were consistent with those of the primary analysis, supporting the robustness of our findings. However, although this landmark analysis reduced the potential for immortal time bias, the exclusion of participants who experienced early CVD events may have introduced a healthy survivor bias, which should be considered when interpreting these sensitivity analyses.

In addition, several covariates showed evidence of non-proportional hazards in the fully adjusted Cox models. Variable-specific tests did not show evidence of violation for the primary HV exposures, including HV transition patterns, cumulative HV, and CHV quartiles (all *P* > 0.05). We therefore performed stratified Cox sensitivity analyses by sex, education, race, BMI category, and smoking status. After stratification, no evidence of violation of the proportional hazards assumption was observed in the HV transition, cumulative HV, and CHV quartile models. The associations were also largely consistent with the primary findings (Supplementary Table S11), supporting the robustness of the observed associations. Nevertheless, these analyses cannot completely exclude the possibility that the magnitude of the HV-CVD associations varied over follow-up time. Future studies with more frequent assessments and time-updated analyses are needed to better characterize the temporal relationship between HV changes and CVD risk.

Second, selection bias and the restricted age range (40–69 years) of the UK Biobank may limit generalizability. Compared with excluded participants, included participants were younger, more educated, had lower BMI and diabetes prevalence, and differed in lifestyle and metabolic characteristics from excluded participants (Supplementary Table S2), indicating a healthier subgroup that could bias associations. Future studies should be validated in broader and more diverse populations.

Third, although HV was constructed using multidomain indicators based on predefined criteria, external validation and formal reliability assessments were not performed. Furthermore, grouping individuals with different vulnerability levels within broader HV transition categories may introduce potential heterogeneity. Future studies with larger samples should explore more refined classification strategies to better capture vulnerability changes. In addition, neuroticism, one of the HV components, was categorized using the sample mean as the cutoff because no established clinical threshold was available for this indicator. However, sensitivity analyses using alternative neuroticism cut-offs were not performed, and different threshold definitions may influence the classification of HV and subsequent transition patterns. Future studies should evaluate the robustness of HV classification using alternative approaches for defining neuroticism and validate the framework in independent populations.

Finally, although we adjusted for multiple demographic, lifestyle, and clinical factors, residual confounding cannot be completely excluded. Specifically, socioeconomic deprivation, blood pressure-related measures, cardiovascular medication use, and kidney disease status were not included as covariates because the variables included in the multivariable models were selected based on established cardiovascular risk factors and the analytical framework of this study. In addition, some of these factors may overlap with aspects captured by HV or potentially reflect health conditions closely related to vulnerability status, and their inclusion may introduce concerns regarding overadjustment. Socioeconomic disadvantage and lower subjective social status have been shown to contribute to cardiovascular disparities and may interact with health vulnerability to influence CVD risk (44–46). Nevertheless, these factors may be associated with both HV and CVD risk, and residual confounding from unadjusted factors cannot be excluded. The direction and magnitude of such residual confounding cannot be precisely quantified. Future studies incorporating more comprehensive cardiovascular risk profiles and causal modeling approaches are needed to further elucidate the relationship between HV transitions and CVD.

## 5 Conclusion

In conclusion, longitudinal changes in HV were associated with subsequent CVD risk in the UK Biobank population. Individuals with increasing or persistently elevated vulnerability exhibited higher risks of CVD compared with those with persistently low vulnerability. These findings suggest that monitoring multidimensional vulnerability transitions may provide additional information for cardiovascular risk assessment. Future studies are needed to elucidate the underlying mechanisms and determine whether interventions targeting health vulnerability changes can effectively reduce CVD risk.

## Data Availability

Publicly available datasets were analyzed in this study. This data can be found here: All data used in this study can be download from public UK Biobank Resource (https://www.ukbiobank.ac.uk/) via application. This study is conducted under application number 98124 for UK Biobank Resource.
