# Supplementary material for: Longitudinal associations of dynamic transitions of multidimensional health vulnerability with cardiovascular disease risk: evidence from UK Biobank
Source: Front Public Health. 2026 Sep 22;14:1902925. doi: 10.3389/fpubh.2026.1902925 (PMC13638419; doi:10.3389/fpubh.2026.1902925)
Supplement: Supplementary file 1 [file Supplementary_File_1.docx]

**Supplement File**

**Supplementary Methods 1. Missing data handling and multiple imputation**

Missing covariates were handled using multiple imputation by chained equations (MICE) with predictive mean matching (PMM), implemented using the R package mice. Five imputed datasets were generated using a fixed random seed (seed = 123) and a maximum of 20 iterations (maxit = 20) under the missing-at-random assumption. Estimates from the five imputed datasets were combined using Rubin’s rules. Detailed information on the distribution of missing data is provided in Supplementary Tables S12-S15.

Participants with missing HV indicators were excluded because complete information was required to calculate the cumulative health vulnerability index and define HV transition patterns. No participant was excluded based on the extent of missingness in covariates, and no prespecified threshold for the number of missing covariates was applied. Among the 16,072 participants in the analytical cohort, 2,874 (17.9%) had at least one missing covariate, while 13,198 (82.1%) had no missing covariates. The mean number of missing covariates per participant was 0.38 (range, 0-6), corresponding to 6,109 missing covariate values in total (Supplementary Tables S12 and S13).

The imputation model included the covariates used in the multivariable analyses, including demographic characteristics, lifestyle factors, diabetes status, BMI, lipid profiles, race, and CVD family history. Survival time and CVD event status were included as auxiliary variables to improve the imputation model. The primary exposure variables, including HV transition patterns and cumulative HV, were not imputed because they were derived from complete HV assessments and were used to define the primary exposure.

PMM was used for continuous variables, logistic regression for binary variables, and multinomial logistic regression for categorical variables with more than two categories. The variables and corresponding imputation methods are summarized in Supplementary Table S14.

The convergence of the MICE procedure was assessed using trace plots of the imputed variables across iterations, with no evidence of substantial non-convergence observed (Supplementary Figure S1). The plausibility of the imputed values was assessed by comparing the distributions of observed and imputed continuous variables (Supplementary Figure S2) and by examining the stability of categorical variable distributions across the five imputed datasets (Supplementary Table S9). The distributions of the imputed values were generally comparable with those of the observed values, supporting the plausibility of the imputation procedure.

## **Supplementary Methods 2. UK Biobank field identification and variable derivation**

All variables were obtained from the corresponding UK Biobank data fields. Health vulnerability (HV) was constructed using 15 binary indicators covering psychological, biological, and social domains. Each indicator was coded as 0 (absence of vulnerability) or 1 (presence of vulnerability) according to predefined criteria.

HV was assessed at baseline (2006-2010) and repeat assessment (2014), and latent classes were identified based on these indicators using latent class analysis.

Covariates included demographic, socioeconomic, lifestyle, anthropometric, and metabolic factors. Age was calculated from year of birth. Education, smoking status, and alcohol consumption were categorized based on UK Biobank definitions and self-reported information.

| Type | Domain | Indicators | UKB field ID |
| --- | --- | --- | --- |
| HV Index | Psychological | Neuroticism score（N-12） | 1920, 1930, 1940, 1950, 1960, 1970, 1980, 1990, 2000, 2010, 2020, 2030 |
|  | Psychological | Current depressive symptoms（RDS-4） | 2050, 2060, 2070, 2080 |
|  | Social | Health satisfaction | 4548 |
|  | Social | Happiness | 4526 |
|  | Psychological | Consultation for anxiety/depression | 2090 |
|  | Biological | Long-standing illness/disability | 2188 |
|  | Biological | Self-rated health | 2178 |
|  | Biological | Falls or operations | 2296, 2415, 2844 |
|  | Biological | Pain | 6159 |
|  | Biological | Physical activity limitation | 6164 |
|  | Biological | Hearing/eye problems | 2247, 6148 |
|  | Social | Friend/family visits | 1031 |
|  | Social | Ability to confide | 2110 |
|  | Social | Leisure/social activities | 6160 |
|  | Social | Number in household | 709 |
| Covariant | Demographics | Age | 34 |
|  | Demographics | Sex | 31 |
|  | Socioeconomic | Education | 6138 |
|  | Lifestyle | Smoking status | 20116 |
|  | Lifestyle | Alcohol drinker status | 20117 |
|  | Anthropometric | BMI | 21001, 23104 |
|  | Biochemical | Total cholesterol | 30690 |
|  | Biochemical | Triglycerides | 30870 |
|  | Biochemical | HDL cholesterol | 30760 |
|  | Biochemical | LDL cholesterol | 30780 |
|  | Comorbidity | Diabetes | 2443 |
|  | Socioeconomic | Race | 21000 |
|  | Socioeconomic | Family history of CVD | 20107 |
| Outcome | CHD | Coronary heart disease | 131296, 131298, 42000 |
|  | Stroke | Stroke | 42006, 131368, 42008, 42010, 131362, 42012, 131360 |
|  | Valvular | Valvular heart disease | 131272, 131276, 131278, 131280, 131284, 131322, 131324, 131326 |
|  | CIHD | Chronic ischemic heart disease | 131306 |
|  | HTN | Hypertensive disease | 131286 |
|  | Arrhythmia | Atrial fibrillation/flutter | 131350 |
|  | Heart failure | Heart failure | 131354 |
|  | Other CVD | The composite other cardiovascular disease category included hypertensive heart disease (HHD), pulmonary heart disease (PHD), myocarditis (MC), cardiomyopathy (CMP), aortic aneurysm (AA), peripheral arterial disease (PAD)/atherosclerosis (AS), and endocarditis (EC). | 131288, 131310, 131334, 131336, 131338, 131340, 131382, 131380, 131330, 131332 |

**Supplementary Methods 3. Assessment of the proportional hazards assumption and stratified Cox sensitivity analyses**

The proportional hazards assumption was assessed using Schoenfeld residual tests based on the cox.zph function in R. Global and variable-specific tests were performed for the Cox regression models, including unadjusted, minimally adjusted, and fully adjusted models. The corresponding results are presented in Supplementary Table S5.

In Model 2 and Model 3, global Schoenfeld tests indicated evidence of proportional hazards violations. Variable-specific analyses showed that these deviations were mainly attributable to covariates, including sex, education, smoking status, BMI, and race, whereas the primary exposure variables did not show evidence of proportional hazards violations. Specifically, the Schoenfeld residual tests for HV transition patterns, cumulative HV, and CHV quartiles were not statistically significant.

To address the potential impact of these non-proportional covariates on the Cox model estimates, stratified Cox sensitivity analyses were performed. Sex, education, race, BMI category, and smoking status were specified as stratification variables. In the Model 2 specification, age was retained as a covariate, whereas in the fully adjusted Model 3 analyses, age, drinking status, diabetes status, lipid profiles (TC, TG, HDL, and LDL), and CVD family history were retained as covariates.

After stratification, global Schoenfeld tests indicated no evidence of violation of the proportional hazards assumption in Model 2 or in the Model 3 analyses of HV transition patterns, cumulative HV, and CHV quartiles (all global P > 0.05; Supplementary Table S11). The associations between HV and CVD risk remained broadly consistent with the primary analyses, supporting the robustness of the main findings.

**Supplementary Figure S1.** Trace plots of imputed continuous variables across iterations in the multiple imputation procedure

**Supplementary Figure S2.** Distributional comparison between observed and imputed values of continuous covariates

### **Supplementary Table S1**. STROBE checklist for cohort studies

| Section/Topic | Reported page | Description | Location |
| --- | --- | --- | --- |
| Title | 1 | Identify study design | Title |
| Abstract | 1-2 | Structured summary | Abstract |
| Introduction | 2-4 | Background and objectives | Introduction |
| Methods | 4-5 | Design, setting, participants | Methods 2.1 |
| Study size | 4-5 | Sample size determination | Methods 2.1 |
| Variables | 5-8 | Definitions of HV, CVD and covariates | Methods (2.2, 2.3, 2.4) |
| Bias | 23-24 | Selection bias assessment | Discussion |
| Statistical methods | 8-10 | LCA/LTA | Methods 2.5 |
|  | 10-11 | Describe all statistical methods, including those used to control for confounding | Methods 2.5 |
|  | 11 | Explain how missing data were handled | Methods 2.5 |
|  | 12 | Describe sensitivity and/or additional analyses | Methods 2.6 |
| Results | 12–19 | Participant flow and outcomes | Results |
| Discussion | 19–24 | Limitations and interpretation | Discussion |

### **Supplementary Table S2**. Baseline characteristics of the analytical cohort vs excluded participants due to incomplete HV information

| Characteristics | Included participants (n=16072) | Excluded participants (n=481045) | *P* value |
| --- | --- | --- | --- |
| Age, mean (SD) | 54.88 (7.61) | 57.12 (8.16) | <0.001 |
| TG, mean (SD)) | 1.61 (0.95) | 1.75 (1.03) | <0.001 |
| TC, mean (SD) | 5.84 (1.05) | 5.69 (1.15) | <0.001 |
| HDL-C, mean (SD) | 1.52 (0.38) | 1.45 (0.38) | <0.001 |
| LDL-C, mean (SD) | 3.65 (0.81) | 3.56 (0.87) | <0.001 |
| BMI, mean (SD) | 26.13 (3.98) | 27.46 (4.80) | <0.001 |
| Sex, n (%) |  |  | 0.198 |
| Female | 8,855 (55.1) | 261,520 (54.6) |  |
| Male | 7,217 (44.9) | 217,644 (45.4) |  |
| Education level, n (%) |  |  | <0.001 |
| Below | 3,947 (24.6) | 195,827 (41.1) |  |
| Higher | 12,125 (75.4) | 280,575 (58.9) |  |
| Smoking status, n (%) |  |  | <0.001 |
| Never | 10,043 (62.5) | 262,559 (54.7) |  |
| Former | 5,003 (31.1) | 165,928 (34.6) |  |
| Current | 1,026 (6.4) | 51,664 (10.8) |  |
| Alcohol consumption, n (%) |  |  | <0.001 |
| Never | 439 (2.7) | 22,571 (4.7) |  |
| Former | 334 (2.1) | 17,623 (3.7) |  |
| Current | 15,299 (95.2) | 439,949 (91.6) |  |
| Diabetes status, n (%) |  |  | <0.001 |
| Yes | 247 (1.5) | 25,750 (5.4) |  |
| No | 15,825 (98.5) | 454,358 (94.6) |  |
| Family history of CVD, n (%) |  |  | 0.004 |
| Yes | 8,416 (53.3) | 256,441 (54.5) |  |
| No | 7,378 (46.7) | 214,459 (45.5) |  |
| Race, n (%) |  |  | 0.026 |
| Non-White | 746 (4.7) | 24,155 (5.0) |  |
| White | 15,276 (95.3) | 455,009 (95.0) |  |

**Note:** Values are presented as mean (standard deviation) for continuous variables and number (percentage) for categorical variables. *P* values were calculated using independent t tests or chi-square tests, as appropriate.

### **Supplementary Table S3**. Baseline and follow-up LCA item-response probabilities

| Time | Indicator | High vulnerability | Moderate vulnerability | Low vulnerability |
| --- | --- | --- | --- | --- |
| baseline | Overall health assessment | 0.856 | 0.768 | 0.323 |
|  | Illness or disability | 0.989 | 0.978 | 0.719 |
|  | Hearing/eye problems | 0.395 | 0.339 | 0.240 |
|  | Falls or operations | 0.576 | 0.346 | 0.129 |
|  | Pain | 0.982 | 0.961 | 0.870 |
|  | physical activity | 0.712 | 0.641 | 0.548 |
|  | N‐12 | 0.886 | 0.214 | 0.342 |
|  | RDS‐4 | 0.649 | 0.097 | 0.057 |
|  | Seen doctor | 0.985 | 0.942 | 0.050 |
|  | Health satisfaction | 0.999 | 0.921 | 0.785 |
|  | Happiness | 0.839 | 0.465 | 0.418 |
|  | Number in household | 0.993 | 0.987 | 0.970 |
|  | Friend/family visits | 0.920 | 0.853 | 0.815 |
|  | Able to confide | 0.758 | 0.689 | 0.560 |
|  | Leisure/social activities | 0.865 | 0.831 | 0.812 |
| Follow-up | Overall health assessment | 0.837 | 0.738 | 0.275 |
|  | Illness or disability | 0.996 | 0.986 | 0.742 |
|  | Hearing/eye problems | 0.462 | 0.397 | 0.297 |
|  | Falls or operations | 0.618 | 0.38 | 0.153 |
|  | Pain | 0.989 | 0.978 | 0.898 |
|  | physical activity | 0.556 | 0.480 | 0.436 |
|  | N‐12 | 0.870 | 0.196 | 0.297 |
|  | RDS‐4 | 0.725 | 0.151 | 0.045 |
|  | Seen doctor | 0.988 | 0.951 | 0.155 |
|  | Health satisfaction | 0.994 | 0.937 | 0.790 |
|  | Happiness | 0.863 | 0.500 | 0.435 |
|  | Number in household | 0.989 | 0.984 | 0.950 |
|  | Friend/family visits | 0.904 | 0.847 | 0.792 |
|  | Able to confide | 0.785 | 0.735 | 0.559 |
|  | Leisure/social activities | 0.827 | 0.793 | 0.741 |

**Note:** Values represent the probability of endorsing Category=1 for each health vulnerability indicator within each latent class.

**Supplementary Table S4.** Posterior classification probabilities for the most likely latent class membership

|  |  | Average latent class assignment probabilities | | | | Posterior classification probabilities | | |
| --- | --- | --- | --- | --- | --- | --- | --- | --- |
| Time | Most likely latent class | High vulnerability | Moderate vulnerability | Low vulnerability | High vulnerability | | Moderate vulnerability | Low vulnerability |
| baseline | High vulnerability | 0.906 | 0.092 | 0.002 | 0.810 | | 0.170 | 0.020 |
|  | Moderate vulnerability | 0.139 | 0.840 | 0.020 | 0.074 | | 0.924 | 0.002 |
|  | Low vulnerability | 0.026 | 0.144 | 0.829 | 0.140 | | 0.032 | 0.828 |
| Follow-up | High vulnerability | 0.894 | 0.103 | 0.003 | 0.923 | | 0.075 | 0.002 |
|  | Moderate vulnerability | 0.125 | 0.844 | 0.031 | 0.169 | | 0.811 | 0.020 |
|  | Low vulnerability | 0.021 | 0.134 | 0.844 | 0.031 | | 0.183 | 0.786 |

## **Supplementary Table S5.** Test of the proportional hazards assumption using Schoenfeld residuals

| Model | | Chi-square | df | *P*-value |
| --- | --- | --- | --- | --- |
| Model 1 | HV Class | 0.965 | 3 | 0.810 |
|  | Cumulative HV (1-SD) | 2.589 | 1 | 0.108 |
|  | CHV Quartile | 3.901 | 3 | 0.272 |
| Model 2 | HV Class | 13.741 | 5 | 0.017 |
|  | Cumulative HV (1-SD) | 14.810 | 3 | 0.002 |
|  | CHV Quartile | 15.891 | 5 | 0.007 |
| Model 3 | HV Class | 41.348 | 16 | 0.001 |
|  | Cumulative HV (1-SD) | 42.030 | 14 | <0.001 |
|  | CHV Quartile | 42.524 | 16 | <0.001 |

**Note:** Model 1: unadjusted; Model 2: adjusted for age and sex;

Model 3: adjusted for age, sex, education level, smoking status, drinking status, diabetes status, race, CVD family history, BMI, TC, TG, HDL, and LDL.

*P*-values were derived from the global test of Schoenfeld residuals. *P*-values < 0.05 indicate potential violation of the proportional hazards assumption.

## **Supplementary Table S6. Sensitivity analysis using alternative HV transition classification**

| **HV transition category** | **Case, n (%)** | **HR (95%CI)** | ***P*-value** |
| --- | --- | --- | --- |
| Stable low vulnerability | 1563(51.6) | Ref |  |
| Dynamic vulnerability | 112(3.7) | 1.62 (1.33-1.96) | <0.001 |
| Persistently elevated vulnerability | 1358(44.7) | 1.28 (1.19-1.38) | <0.001 |

**Note:** Adjusted for age, sex, education level, smoking status, drinking status, diabetes status, race, CVD family history, BMI, TC, TG, HDL, and LDL.

## **Supplementary Table S7. Sensitivity analysis excluding hypertensive diseases from the composite cardiovascular outcome**

| **HV transition category** | **Cases, n (%)** | **HR (95% CI)** | ***P* value** |
| --- | --- | --- | --- |
| Class 1: Persistently low vulnerability | 633 (55.3) | Ref |  |
| Class 2: Decreasing vulnerability | 8 (0.7) | 1.23 (0.84–1.81) | 0.29 |
| Class 3: Increasing vulnerability | 38 (3.3) | 1.77 (1.42–2.22) | <0.001 |
| Class 4: Persistently elevated vulnerability | 466 (40.7) | 1.30 (1.21–1.41) | <0.001 |

**Note:** Adjusted for age, sex, education level, smoking status, drinking status, diabetes status, race, CVD family history, BMI, TC, TG, HDL, and LDL.

**Supplementary Table S8.** Landmark sensitivity analysis excluding participants with incident CVD between the baseline and repeat health vulnerability assessments

|  | **Variables** | **Case, n (%)** | **Model 1^a^** | | **Model 2^b^** | | **Model 3c** | |
| --- | --- | --- | --- | --- | --- | --- | --- | --- |
|  |  |  | **HR (95%CI)** | ***P* value** | **HR (95%CI)** | ***P* value** | **HR (95%CI)** | ***P* value** |
|  | **Categories** |  |  |  |  |  |  |  |
|  | class1 | 454(54.2) | 1.00 (Ref) |  | 1.00 (Ref) |  | 1.00 (Ref) |  |
|  | class2 | 8(0.9) | 1.26 (0.63-2.54) | 0.511 | 1.62 (0.8–3.25) | 0.179 | 1.52 (0.76-3.07) | 0.239 |
|  | class3 | 14(1.7) | 1.07 (0.63-1.82) | 0.806 | 1.2 (0.7–2.04) | 0.503 | 1.14 (0.67-1.95) | 0.622 |
|  | class4 | 361(43.2) | 1 (0.88-1.15) | 0.946 | 1.22 (1.06–1.41) | 0.005 | 1.18 (1.02-1.36) | 0.025 |
|  | ***P* for trend** |  |  | 0.945 |  | 0.005 |  | 0.026 |
|  | **Cumulative health vulnerability** | | 1.22 (1.13-1.31) | <0.001 | 1.29 (1.19–1.39) | <0.001 | 1.23 (1.14-1.33) | <0.001 |
|  | **Quartiles** |  |  |  |  |  |  |  |
|  | Q1(<19.0) | 224(26.8) | 1.00 (Ref) |  | 1.00 (Ref) |  | 1.00 (Ref) |  |
|  | Q2(19.0-30.1) | 225(26.9) | 1.12 (0.93-1.35) | 0.227 | 1.15 (0.95-1.38) | 0.142 | 1.11 (0.92-1.33) | 0.280 |
|  | Q3(30.1-44.2) | 218(26.1) | 1.31 (1.09-1.58) | 0.005 | 1.41 (1.17-1.7) | <0.001 | 1.33 (1.1-1.61) | 0.003 |
|  | Q4(≥44.2) | 170(20.2) | 1.51 (1.23-1.85) | 0.000 | 1.72 (1.41-2.11) | <0.001 | 1.54 (1.25-1.89) | <0.001 |
|  | ***P* for trend** |  |  | <0.001 |  | <0.001 |  | <0.001 |
| **Note:** In this landmark sensitivity analysis, the date of the repeat HV assessment in 2014 was used as the time origin for CVD follow-up. Participants who developed CVD or hypertension between the baseline and repeat HV assessments were excluded. Although this approach reduced the potential for immortal time bias, the exclusion of participants with early CVD events may have introduced healthy survivor bias.  **^a^Model 1** unadjusted.  **^b^Model 2** adjusted for age, sex.  **^c^Model 3** further adjusted for education level, smoking status, drinking status, diabetes status, race, CVD family history, BMI, TG, TC, HDL, LDL on model 2. | | | | | | | | |

### **Supplementary Table S9. Stability of categorical variables across imputed datasets**

| **Variable** | **Category** | **Original data** | **Imputation 1** | **Imputation 2** | **Imputation**  **3** | **Imputation 4** | **Imputation 5** |
| --- | --- | --- | --- | --- | --- | --- | --- |
| **Race** | White | 15,276 (95.3%) | 15,323 (95.3%) | 15,323 (95.3%) | 15,324 (95.3%) | 15,324 (95.3%) | 15,322 (95.3%) |
|  | Non-white | 746 (4.7%) | 749 (4.7%) | 749 (4.7%) | 748 (4.7%) | 748 (4.7%) | 750 (4.7%) |
| **CVD family history** | No | 7,378 (46.7%) | 7,508 (46.7%) | 7,514 (46.8%) | 7,497 (46.6%) | 7,503 (46.7%) | 7,515 (46.8%) |
|  | Yes | 8,416 (53.3%) | 8,564 (53.3%) | 8,558 (53.2%) | 8,575 (53.4%) | 8,569 (53.3%) | 8,557 (53.2%) |

## **Supplementary Table S10. Variable-specific Schoenfeld residual tests for the proportional hazards assumption**

|  |  | **Model 2** | | | **Model 3** | | |
| --- | --- | --- | --- | --- | --- | --- | --- |
| **Model** | **Variable** | **Chi-square** | **df** | **P-value** | **Chi-square** | **df** | ***P*-value** |
| Class |  |  |  |  |  |  |  |
|  | class (overall) | 0.991 | 3 | 0.803 | 1.042 | 3 | 0.791 |
|  | age | 3.127 | 1 | 0.077 | 3.592 | 1 | 0.058 |
|  | **sex** | 8.504 | 1 | 0.004 | 8.892 | 1 | **0.003** |
|  | **education** | — | — | — | 5.007 | 1 | **0.025** |
|  | **smoking** | — | — | — | 4.091 | 1 | **0.043** |
|  | drinking | — | — | — | 0.859 | 1 | 0.354 |
|  | diabetes | — | — | — | 0.026 | 1 | 0.871 |
|  | **BMI** | — | — | — | 7.219 | 1 | **0.007** |
|  | TC | — | — | — | 1.169 | 1 | 0.280 |
|  | TG | — | — | — | 0.081 | 1 | 0.776 |
|  | HDL | — | — | — | 1.675 | 1 | 0.196 |
|  | LDL | — | — | — | 0.802 | 1 | 0.371 |
|  | **race** | — | — | — | 6.078 | 1 | **0.014** |
|  | CVD family history | — | — | — | 0.124 | 1 | 0.725 |
| Cumulative HV (1-SD) |  |  |  |  |  |  |  |
|  | std_cum_hv | 2.761 | 1 | 0.097 | 2.796 | 1 | 0.095 |
|  | age | 3.198 | 1 | 0.074 | 3.646 | 1 | 0.056 |
|  | **sex** | 8.443 | 1 | **0.004** | 8.867 | 1 | **0.003** |
|  | **education** | — | — | — | 5.051 | 1 | **0.025** |
|  | **smoking** | — | — | — | 4.089 | 1 | **0.043** |
|  | drinking | — | — | — | 0.845 | 1 | 0.358 |
|  | diabetes | — | — | — | 0.018 | 1 | 0.895 |
|  | **BMI** | — | — | — | 7.202 | 1 | **0.007** |
|  | TC | — | — | — | 1.197 | 1 | 0.274 |
|  | TG | — | — | — | 0.096 | 1 | 0.756 |
|  | HDL | — | — | — | 1.674 | 1 | 0.196 |
|  | LDL | — | — | — | 0.820 | 1 | 0.365 |
|  | **race** | — | — | — | 6.048 | **1** | **0.014** |
|  | CVD family history | — | — | — | 0.116 | 1 | 0.734 |
| Quartile |  |  |  |  |  |  |  |
|  | cum_hv_group (overall) | 3.730 | 3 | 0.292 | 3.318 | 3 | 0.345 |
|  | age | 3.192 | 1 | 0.074 | 3.634 | 1 | 0.057 |
|  | **sex** | 8.514 | 1 | **0.004** | 8.922 | 1 | **0.003** |
|  | **education** | — | — | — | 5.035 | 1 | **0.025** |
|  | **smoking** | — | — | — | 4.072 | 1 | **0.044** |
|  | drinking | — | — | — | 0.836 | 1 | 0.361 |
|  | diabetes | — | — | — | 0.019 | 1 | 0.890 |
|  | **BMI** | — | — | — | 7.266 | 1 | **0.007** |
|  | TC | — | — | — | 1.200 | 1 | 0.273 |
|  | TG | — | — | — | 0.090 | 1 | 0.764 |
|  | HDL | — | — | — | 1.685 | 1 | 0.194 |
|  | LDL | — | — | — | 0.827 | 1 | 0.363 |
|  | **race** | — | — | — | 5.954 | 1 | **0.015** |
|  | CVD family history | — | — | — | 0.117 | 1 | 0.732 |

**Note:** Model 2 was adjusted for age and sex, whereas Model 3 was fully adjusted.*P*-values in bold indicate evidence of a potential violation of the proportional hazards assumption (*P* < 0.05).For categorical variables with more than two levels (class and cumulative HV quartiles), the global Schoenfeld residual test is reported.

## **Supplementary Table S11. Sensitivity analysis using stratified Cox models accounting for violations of the proportional hazards assumption**

### **Part A. Associations of HV transitions, cumulative HV, and CHV quartiles with CVD risk**

| **Analysis** | **Exposure / Comparison** | **HR (95% CI)** | ***P* value** |
| --- | --- | --- | --- |
| HV transition patterns | Class 2 vs Class 1 | 1.27 (0.86–1.89) | 0.231 |
|  | **Class 3 vs Class 1** | **1.84 (1.48–2.30)** | **<0.001** |
|  | **Class 4 vs Class 1** | **1.29 (1.20–1.39)** | **<0.001** |
| Cumulative HV | **Per 1-SD increase** | **1.17 (1.14–1.22)** | **<0.001** |
| CHV quartiles | Q2 vs Q1 | 1.15 (1.03–1.28) | 0.011 |
|  | **Q3 vs Q1** | **1.24 (1.11–1.38)** | **<0.001** |
|  | **Q4 vs Q1** | **1.54 (1.38–1.70)** | **<0.001** |

**Note:** Stratified Cox models included sex, education, race, BMI category, and smoking status as stratification variables. These variables were selected based on variable-specific Schoenfeld residual tests indicating potential non-proportionality (*P*<0.05). BMI was categorized as <24 kg/m² and ≥24 kg/m² for stratification purposes. For the Model 2 specification, age was included as a covariate. For the fully adjusted Model 3 analyses, age, drinking status, diabetes status, lipid profiles (TC, TG, HDL, and LDL), and CVD family history were included as covariates.

**Part B. Proportional hazards assumption after stratification**

| **Model** | **Global χ²** | **df** | ***P* value** |
| --- | --- | --- | --- |
| HV transition patterns model (Model 2 specification) | 5.96 | 4 | 0.203 |
| HV transition patterns model (Model 3, fully adjusted) | 11.26 | 11 | 0.422 |
| Cumulative HV model | 10.97 | 9 | 0.278 |
| CHV quartiles model | 11.57 | 11 | 0.397 |

**Note:** Schoenfeld residual tests indicated no evidence of violation of the proportional hazards assumption after stratification in Model 2 or Model 3 (all global *P* > 0.05). For the HV transition patterns model, the variable-specific test for the HV class variable was also non-significant (χ² = 2.79, df = 3, *P* = 0.426).

## **Supplementary Table S12. Missing data distribution across covariates**

|  | **Variable** | **Missing, n (%)** |
| --- | --- | --- |
|  | Sex | 0 (0.00) |
|  | Age | 0 (0.00) |
|  | Education level | 58 (0.36) |
|  | Smoking status | 33 (0.21) |
|  | Drinking status | 4 (0.02) |
|  | Diabetes status | 25 (0.16) |
|  | BMI | 243 (1.51) |
|  | TC | 1,025 (6.38) |
|  | TG | 1,041 (6.48) |
|  | HDL | 2,815 (17.51) |
|  | LDL | 1,064 (6.62) |
|  | Race | 50 (0.31) |
|  | CVD family history | 278 (1.73) |

**Note:** Percentages were calculated based on the analytical cohort (n = 16,072).

## **Supplementary Table S13. Number of missing covariates per participant**

| **Number of missing covariates** | **Participants, n (%)** |
| --- | --- |
| 0 | 13,198 (82.12) |
| 1 | 1,739 (10.82) |
| 2 | 99 (0.62) |
| 3 | 12 (0.07) |
| 4 | 988 (6.15) |
| 5 | 32 (0.20) |
| 6 | 4 (0.02) |

**Note:** Percentages were calculated based on the analytical cohort (n = 16,072). The mean number of missing covariates per participant was 0.38 (range: 0–6).

**Supplementary Table S14. Variables included in the multiple imputation model and imputation methods used**

| **Variable category** | **Variables** | **Imputation method / role** |
| --- | --- | --- |
| Continuous | Age | IiII Includ in imputation model; not imputed because no missing values |
| Continuous | BMI, TC, TG, HDL, LDL | Predictive mean matching (PMM) |
| Binary | Sex, diabetes status, race, CVD family history, education level | Logistic regression |
| Categorical (>2 categories) | Smoking status, drinking status | Multinomial logistic regression |
| Auxiliary (not imputed) | Survival time, CVD event status | Included to improve imputation accuracy |
| Not imputed (primary exposure) | HV transition patterns, cumulative HV | Derived from complete HV assessments; not imputed |
| Not imputed (identifier) | n_eid | Participant identification only |

**Note:** The MICE procedure was conducted under the assumption that missingness was at random conditional on the variables included in the imputation model.See Supplementary Methods 1 for details on the imputation procedure, and Supplementary Table S12 for missing data proportions by variable.

### **Supplementary Table S15. Summary of missing data and multiple imputation**

| **Summary measure** | **Value** |
| --- | --- |
| Analytical cohort, n | 16,072 |
| Participants with no missing covariates, n (%) | 13,198 (82.1) |
| Participants with ≥1 missing covariate, n (%) | 2,874 (17.9) |
| Total missing covariate values, n | 6,109 |
| Mean number of missing covariates per participant | 0.38 |
| Range of missing covariates per participant | 0–6 |
| Pre-specified threshold for exclusion based on covariate missingness | None applied |
| Number of imputed datasets | 5 |
| Participants excluded due to missing HV indicators | 331,116 (65.9%)* |

**Note:** Percentages for the analytical cohort are based on n = 16,072. Missing covariates were handled using MICE, and all missing covariate values were eligible for imputation; no participant was excluded based on the number of missing covariates. Participants with missing HV indicators were excluded because complete HV information was required to calculate the cumulative HV index and define HV transition patterns. *The denominator for this percentage (502,370) is the initial screened population before exclusions, which differs from the analytical cohort (16,072).
